# Identification and validation of tryptophan metabolism-related lncRNAs in lung adenocarcinoma prognosis and immune response

**DOI:** 10.1007/s00432-024-05665-x

**Published:** 2024-04-01

**Authors:** Mingjun Gao, Mengmeng Wang, Yong Chen, Jun Wu, Siding Zhou, Wenbo He, Yusheng Shu, Xiaolin Wang

**Affiliations:** 1 Dalian Medical University, Dalian, 116000 China; 2 Clinical Medical College, Yangzhou University, Yangzhou, 225000 China; 3 Department of Thoracic Surgery, Northern Jiangsu People’s Hospital, No. 98 Nantong West Road, Yangzhou, 225000 Jiangsu China

**Keywords:** Lung adenocarcinoma, Tryptophan metabolism, LncRNA, Prognosis, Immune

## Abstract

**Background:**

Tryptophan (Trp) is an essential amino acid. Increasing evidence suggests that tryptophan metabolism plays a complex role in immune escape from Lung adenocarcinoma (LUAD). However, the role of long non-coding RNAs (lncRNAs) in tryptophan metabolism remains to be investigated.

**Methods:**

This study uses The Cancer Genome Atlas (TCGA)-LUAD dataset as the training cohort, and several datasets from the Gene Expression Omnibus (GEO) database are merged into the validation cohort. Genes related to tryptophan metabolism were identified from the Molecular Signatures Database (MSigDB) database and further screened for lncRNAs with Trp-related expression. Subsequently, a prognostic signature of lncRNAs related to tryptophan metabolism was constructed using Cox regression analysis, (Least absolute shrinkage and selection operator regression) and LASSO analysis. The predictive performance of this risk score was validated by Kaplan–Meier (KM) survival analysis, (receiver operating characteristic) ROC curves, and nomograms. We also explored the differences in immune cell infiltration, immune cell function, tumor mutational load (TMB), tumor immune dysfunction and exclusion (TIDE), and anticancer drug sensitivity between high- and low-risk groups. Finally, we used real-time fluorescence quantitative PCR, CCK-8, colony formation, wound healing, transwell, flow cytometry, and nude mouse xenotransplantation models to elucidate the role of ZNF8-ERVK3-1 in LUAD.

**Results:**

We constructed 16 tryptophan metabolism-associated lncRNA prognostic models in LUAD patients. The risk score could be used as an independent prognostic indicator for the prognosis of LUAD patients. Kaplan–Meier survival analysis, ROC curves, and risk maps validated the prognostic value of the risk score. The high-risk and low-risk groups showed significant differences in phenotypes, such as the percentage of immune cell infiltration, immune cell function, gene mutation frequency, and anticancer drug sensitivity. In addition, patients with high-risk scores had higher TMB and TIDE scores compared to patients with low-risk scores. Finally, we found that ZNF8-ERVK3-1 was highly expressed in LUAD tissues and cell lines. A series of in vitro experiments showed that knockdown of ZNF8-ERVK3-1 inhibited cell proliferation, migration, and invasion, leading to cell cycle arrest in the G0/G1 phase and increased apoptosis. In vivo experiments with xenografts have shown that knocking down ZNF8-ERVK3-1 can significantly inhibit tumor size and tumor proliferation.

**Conclusion:**

We constructed a new prognostic model for tryptophan metabolism-related lncRNA. The risk score was closely associated with common clinical features such as immune cell infiltration, immune-related function, TMB, and anticancer drug sensitivity. Knockdown of ZNF8-ERVK3-1 inhibited LUAD cell proliferation, migration, invasion, and G0/G1 phase blockade and promoted apoptosis.

**Supplementary Information:**

The online version contains supplementary material available at 10.1007/s00432-024-05665-x.

## Background

Lung cancer (LC) is one of the most common malignancies worldwide, and lung adenocarcinoma is the most common pathological type, accounting for nearly 40% of all lung cancer subtypes (Sung et al. 2021; Lortet-Tieulent et al. 2014). In recent decades, the development of molecularly targeted therapies and immune checkpoint inhibitors (ICIs) has improved the survival of LUAD patients. However, LUAD survival remains poor, with a 5-year survival rate of less than 20% (Hirsch et al. 2017). Therefore, developing an effective prognostic model to predict LUAD prognosis is crucial.

Tryptophan is an essential amino acid. Increasing evidence suggests that tryptophan metabolism is involved in tumor development through various mechanisms. Trp metabolism has a complex and multifaceted role in immune escape from LC cells and cancer-associated cells (Godin-Ethier et al. 2011). Trp is degraded via the kynurenine (KYN) pathway and metabolized to form serotonin or other metabolites (Schwarcz and Stone 2017). Indoleamine 2,3-dioxygenase 1 (IDO1) and tryptophan 2,3-dioxygenase (TDO) catalyze the first and rate-limiting steps of tryptophan metabolism (Cervenka et al. 2017). IDO1 inhibitors have been used in clinical trials for cancer immunotherapy and have emerged as a critical target for tumor immunotherapy. IDO1 blockers are used with ICIs to inhibit tumor growth (Yentz and Smith 2018). The IDO1 inhibitor, Epacadostat, shows potent anti-IDO1 activity by promoting T-cell activation and inhibiting regulatory T-cell function (Komiya and Huang 2018; Jochems et al. 2016). Recent studies have shown that IDO1-associated Try metabolites are strongly associated with the development of lung cancer (Yoshida et al. 1981). It has been demonstrated that increased IDO1 activity was detected in lung cancer patients with recurrent metastases after receiving immunotherapy (Agulló-Ortuño et al. 2020). LC cells with high expression of IDO1 have enhanced invasive ability in vitro and distant metastasis to the brain, liver, and bone in vivo, whereas IDO1 inhibition attenuates their invasion and distant metastasis (Tang et al. 2017). Similarly, IDO1 inhibition suppresses lung metastasis in breast cancer (Smith et al. 2012; Levina et al. 2012). Tryptophan metabolism is a key target for tumor immunotherapy. Therefore, a comprehensive analysis of the Trp pathway may improve survival and provide a potential strategy for precise treatment of LUAD patients.

lncRNAs are a class of RNA molecules with transcripts longer than 200 nt (Volders et al. 2019). In many cancers, lncRNAs are aberrantly expressed and regulate biological properties such as cell proliferation, cell invasion, and cell cycle (Chen et al. 2017). Various studies have shown that lncRNAs are implicated in tumorigenesis, disease progression, and drug resistance in LUAD (Feng et al. 2021; Chen et al. 2021; Yu et al. 2020). Down-regulation of the lncRNA LHFPL3-AS2 reduces its specific interaction with SFPQ, leading to more SFPQ binding to the TXNIP promoter, causing transcriptional repression of TXNIP, which ultimately promotes metastasis in non-small cell lung cancer (Cheng et al. 2022). In addition, M2 macrophage-derived exosomal lncRNA AGAP2-AS1 enhances immunity to radiotherapy in lung cancer by decreasing miR-296 and elevating NOTCH2 (Zhang et al. 2021).

Currently, studies on the role of Trp metabolism-associated lncRNA genes and LUAD patients still need to be made available. Therefore, potential Trp metabolism-associated lncRNAs were mined based on the TCGA-LUAD cohort to construct a risk model for the prognosis of LUAD patients. The applicability of this risk profile has been validated in the GEO cohort. The mechanism of Trp metabolism-associated lncRNAs in lung adenocarcinoma and TME heterogeneity were explored by functional enrichment and immune infiltration analyses. The risk model also helped to differentiate the response of LUAD patients to targeted therapeutic and chemotherapeutic agents. Finally, we also preliminarily verified the mechanism by which ZNF8-ERVK3-1 promotes lung adenocarcinoma development by in vitro cellular experiments. In this study, we attempted to elucidate the clinical value of Trp metabolism-associated lncRNAs in LUAD patients using bioinformatics analysis to provide theoretical support for individualized treatment of LUAD patients.

## Methods

### Data acquisition

RNA sequencing data and corresponding clinical information were extracted from the TCGA database for 516 patients with LUAD. 9 patients were excluded due to lack of available survival information, and 507 patients with LUAD were included for further analysis. Ensembl IDs were then converted to official gene symbols, and the data were log2 processed. Mutation data for the included LUAD patients were also obtained from TCGA. These patients were randomly divided into a training group (*n* = 253) and a test group (*n* = 254) in a 1:1 ratio using the "caret" R package (Kuhn 2008). No statistically significant difference was found in clinical features between the training group and the test group (*p* > 0.05).

lncRNAs and mRNAs were isolated by sorting the downloaded transcriptome data using Perl software. Ethics committee approval was not required as the clinical information of the patients participating in this study was obtained from the TCGA database, and the TCGA publication guidelines were strictly adhered to. We included four external datasets with lncRNA expression profiles and prognostic information to validate the models: the GSE30219 (85 LUAD samples), the GSE37745 (106 LUAD samples), the GSE50081 (127 LUAD samples), and the GSE31210 (226 LUAD samples). In the GEO database, all files were received from the platform with the GPL570 standard, and all data were log2 transformed. The software package "SVA" was used to run the algorithm cbind function to integrate the four datasets and the ComBat function to remove batch effects and normalize. A final cohort of 544 LUAD patients will be included in the GEO validation cohort.

### Identification of tryptophan metabolism-related lncRNAs

We extracted the genes involved in the tryptophan metabolic pathway "KEGG tryptophan metabolism" from the MSigDB database (https://www.gsea-msigdb.org/gsea/msigdb) of Kyoto Encyclopedia of Genes and Genomes (KEGG) (Liberzon et al. 2015), and a total of 59 genes were extracted (Table S1). The association coefficients between lncRNAs and Trp-related genes were calculated using the R software "limma" package. The screening criteria were |cor|> 0.4 and P-value < 0.001. Sankey diagrams were obtained using the R package "galluvial".

### Model construction

To identify lncRNA profiles associated with tryptophan metabolism, we first used univariate Cox analysis to screen for lncRNAs associated with LUAD overall survival. Then LASSO analysis was performed for further screening (Tibshirani 1997). Finally, multivariate Cox regression analysis was finalized, identifying of 16 lncRNAs associated with tryptophan metabolism for a more robust prognostic risk model. Patients in the training cohort were categorized into low-risk and high-risk groups based on the median risk score. The risk score was calculated using the following formula:

$${\text{risk score}} = \sum \exp \;\ln {\text{cRNA}}_{i} \times \beta_{i} ,$$

where exp lncRNAi is the relative expression of tryptophan metabolism-related lncRNAs and βi is the regression coefficient.

### Risk assessment models

The test cohort and the whole cohort were used for model validation, and the risk score algorithm was used to calculate each person's risk score. Subjects were assigned to a high-risk group (risk score above threshold) or a low-risk group (risk score below threshold) based on a threshold (median risk score). Overall survival (OS) and progression-free survival (PFS) were analyzed using the Kaplan–Meier method. The PFS dataset was obtained from UCSC Xena (http://xena.ucsc.edu/). Univariate and multivariate Cox regression analyses were used to investigate whether the risk model was an independent risk factor excluding other clinical characteristics (age, sex, and stage). Heatmaps of patient survival status and expression of lncRNAs based on risk scores were plotted using the pheatmap software package. The "timeROC" R software package was used to perform ROC analyses and area under the curve (AUC) calculations, and the c-index curves were plotted using the R software "survival", "rms", and "pec" packages to evaluate the predictive ability of prognostic characteristics. Principal component analysis (PCA) was used to assess the ability to group genome-wide, tryptophan metabolism genes, all tryptophan lncRNAs, and tryptophan lncRNA features of the risk model. Patients were categorized into stages I–II and III–IV to determine the suitability of risk profiles for patients with different stages of LUAD.

### Construction of forecast line charts

Clinicopathological factors were combined with our constructed risk scores to construct column charts for predicting 1-, 3-, and 5-year OS rates in LUAD patients. The "rms" R package was used to construct predictive column charts and corresponding calibration curves. The closer the calibration curve is to the diagonal, the better the prognostic predictive performance of the column chart.

### Validation of risk models

The risk score for each case in the GEO cohort was calculated using the formula used in the TCGA cohort. Using the median risk score from the TCGA cohort as a criterion for risk setting in the GEO cohort, we assigned 272 cases to the low-risk group. The remaining 272 cases were included in the high-risk group. The risk model was analyzed using a similar approach in the TCGA-LUAD training set to determine whether it was an independent prognostic factor in the validation cohort. The validity of predicting prognosis was validated using KM curves to assess survival outcomes in the risk group in the GEO validation dataset. The predictive power of prognostic features was calculated by ROC analysis. Univariate and multivariate Cox regression analyses were performed to investigate whether the risk model was an independent risk factor excluding other clinical characteristics (age, sex, and stage).

### Functional enrichment analysis

Differentially expressed genes (DEGs) were analyzed between high- and low-risk groups using the "limma "R package, |log2FC|> 1 and False Discovery Rate (FDR) < 0.05.Tryptophan metabolism-associated lncRNAs were analyzed using the clusterProfiler package for Gene Ontology (GO) and KEGG enrichment (Kanehisa and Goto 2000). lncRNAs were analyzed for GO and KEGG enrichment.

### Tumor mutational burden (TMB) analysis and immune-related functional analysis

We downloaded TMB-related data from the TCGA samples and analyzed the number of mutations in both subgroups of LUAD patients using the R package (Maftools package). We used the survival package to determine the difference between the survival of patients with high and low TMB. *P*-values < 0.05 were considered statistically significant. Immunological scores of LUAD patients were calculated by the ESTIMATE algorithm (Yoshihara et al. 2013). Score scores for 22 immune cell subtypes in each tumor sample were identified by CIBERSORT (cell type identification by estimating the relative subtypes of RNA transcripts) (Newman et al. 2015). The relative abundance of different immune cell types in the low and high-risk groups was quantified and assessed to compare and predict immune cell infiltration between the two groups. Correlation analysis of immune function was based on ssGSEA (Subramanian et al. 2005).

### Predicting response to immunotherapy and chemotherapy

NSCLC immune dysfunction and rejection were obtained by TIDE (http://tide.dfci.harvard.edu/). TIDE scores were analyzed using the "limma" and "ggpubr" R packages in the high- and low-risk groups. TIDE scores accurately predicted the efficacy of immunotherapeutic drugs received by patients (Jiang et al. 2018), with higher TIDE scores predicting poor response to immunotherapy. Higher TIDE scores predict poor response to immunotherapy.

The Genomics of Drug Sensitivity in Cancer (GDSC) is a public dataset containing information about drug sensitivity and molecular markers of drug response in cancer cells (Iorio et al. 2016). The "oncoPredict" package was used to predict the drug sensitivity of LUAD samples to various antineoplastic drugs (Maeser et al. 2021).

### Tissue sample collection and lung adenocarcinoma cell line culture

All tissue samples were collected from the Department of Thoracic Surgery of the People's Hospital of Northern Jiangsu Province and approved by the hospital's Medical Ethics Committee. We obtained informed consent from each relevant patient before collection. Sixteen pairs of samples, including tumor tissue (T) and paired normal tissue (N), were obtained from patients with lung adenocarcinoma who underwent tumor resection between January 2019 and January 2022, and the pathological type of all LUAD cases was lung adenocarcinoma. All samples were stored at -80 °C. HBE, A549, H1975, H1299, and PC9 cell lines were obtained from the China Cell Resource Centre (Shanghai, China). Cells were cultured in RPMI 1640 (Solarbio) medium supplemented with 10% fetal bovine serum (Procell). Cells were incubated in a humidified incubator (Thermo Scientific, China) with 5% CO2, 37 °C. The cells were incubated in a humidified incubator (Thermo Scientific, China) with 5% CO2.

### RNA extraction and quantitative real-time polymerase chain reaction (qRT-PCR)

RNA was extracted from tissues and cells using TRIzol reagent (Vazyme). We measured RNA concentration using a spectrophotometer and stored the samples at – 80 °C cDNA was synthesized using the Hifair®III 1st Strand cDNA Synthesis SuperMix for qPCR (gDNA digester plus) (Yeasen Biotechnology, Shanghai, China). Quantitative real-time PCR was performed using Hieff®qPCR SYBR Green Master Mix (High Rox Plus) (Yeasen Biotechnology, Shanghai, China) in StepOne Plus real-time PCR System (Applied Biosystems). The relative expression of ZNF8-ERVK3-1 was normalized to the endogenous control GAPDH using the 2-ΔΔCt method, respectively. The primer sequences were:ZNF8-ERVK3-1:F:′-CAAGCATCACGCAAGGAAGAGG-3′, R:5′- TGGTGGGATAAGGAGCATCTGTC-3′;GAPDH:F:5′-TCATTTCCTGGGACACGA-3′,R:5′-GTCTTACTCCTTGGAGGCC-3′.

### Cell transfection

The siRNA and siRNA negative control (siNC) were purchased from GenePharma (Shanghai, China). siRNA sequences were as follows: ZNF8-ERVK3-1 siNC sense:5′-UUCUCCGAACGUGUCACGUTT -3′, antisense:5′- acgugacacguucagaat -3′; siRNA1 sense:5′-GAAGGUCUGUCCUCGUGUUTT -3′, antisense:5′-AACACGAGGACAGACCUUCTT-3′; siRNA2 sense:5′- GCGAGACUGUGGGAGAACUTT -3′, antisense:5′-AGUUCUCCCACAGUCUCGCTT -3ʹ; siRNA3 sense:5′- GUGACCUGGAACAACAAUATT -3′, antisense:5′- UAUUGUUGUUUCCAGGUCACTT -3′. Cells were incubated in 6-well plates, and transfection was started when cell density reached 60%. Transfection was performed using gp -transfection-mate (GenePharma). Transfection efficiency was detected using qRT-PCR.

### Cell proliferation assay

The cell proliferation assay was performed on a panel of 96 wells, and 1000 cells were added to each well after counting. After 24, 48, 72, and 96 h, 10 μL of CCK-8 solution (Yeasen) was added to each well and incubated for 1 h. Absorbance (OD) at 450 nm was detected in each well by an enzyme labeling instrument (Skanlt RE 7.0).

### Cell migration and invasion assay

We used 8 µm pore size Transwell chambers (Corning, USA) in 24-well plates; 200ul of cell suspension containing 10,000 FBS-free cells was added to the upper chambers with matrix gel (BD Biocoat) or without matrix gel (Corning), and 1640 500 ul containing 10% FBS was added to the lower chambers. The cells were cultured in a cell incubator for 48 h. Cells suspended in the chambers were rinsed with phosphate buffer saline (PBS), cells were fixed with 4% paraformaldehyde for 15 min, stained with 0.1% crystal violet solution for 5 min, washed with PBS three times, and cells on the upper surface of the bottom chamber were gently wiped with a cotton swab. Cells on the lower surface of the bottom of the drying chamber, images were taken with an inverted microscope (OLYMPUS-CKX53). Cell counting was performed using Image J.

### Wound healing test

Wound healing assay was performed to assess the migratory capacity of the cells. Transfected cells (15 × 10^4^/well) were inoculated in six-well plates with a monolayer of cells evenly distributed on the bottom of the plate. The cell layer was scraped with a 200 μl pipette tip, washed with PBS, and FBS-free medium was added to each well. Images were then taken under an inverted microscope at 0 and 48 h (OLYMPUS-CKX53, China). The images were analyzed using Image J software.

### Colony formation assay

siNC and siRNA3 targeting the ZNF8-ERVK3-1 gene were used to transfect cells. 1000 cells were inoculated into each well of a 6-well culture plate and incubated for 2 weeks. 4% paraformaldehyde fixed the cells for 15 min, 0.1% crystal violet solution stained for 10 min, air-dried and photographed, and colonies were counted using Image J software.

### Flow cytometry

Cell cycle: PBS washed 3 times, trypsin digested for 3 min, fixed in 70% ethanol at 4℃ for 30 min, and then incubated in 500 μl of propidium iodide staining solution (PI) (Beyotime) at 37 ℃ for 30 min. Apoptosis: PBS washed 3 times, trypsin digested for 3 min, and then annexin-FITC reagent (Beyotime) was added in order, and incubated for 20 min, protected from light. Cell cycle and apoptosis were detected by flow cytometry (BD Biosciences, USA), and the results were analyzed using flowJo software.

### Immunohistochemical staining (IHC)

Sections of 3 μm thick paraffin-embedded tissues were dewaxed and hydrated, and antigenic repair was performed in pH 6.0 sodium citrate antigen repair solution in a microwave oven for 2 min on high and 15 min on bottom. Peroxidase activity was blocked by incubation in endogenous peroxide blocking solution (Beyotime, code:P0100A) for 25 min. Non-specific staining was blocked by confinement in immunostaining confinement solution (Beyotime, code:P0260) for 15 min. Sections were incubated overnight with anti-PCNA (Cell Signaling, code:13,110, 1:500 dilution) and anti-ki67 (proteintech, code:27,309–1-AP, 1: 2000 dilution). Placed at room temperature, the sections were washed 3 times with PBS, followed by incubation with secondary anti-IgG antibody (Servicebio, product number:G1215-200T) at room temperature for 1 h. DAB was used as a chromogen. The nucleus was stained with hematoxylin solution. The sections were scanned with KScanner software.

### Xenograft model

The Laboratory Animal Ethics Committee of Yangzhou University approved animal experiments. A mouse xenograft model was established to explore the functional role of ZNF8-ERVK3-1 in vivo. H1975 cells were plated in six-well plates and transfected with siNC and siRNA3. Ten BALB/c nude mice from Nanjing Ji Biotechnology Co., Ltd. were randomly divided into two groups, and the above-treated cells (8 × 10^5^) were injected into the axillary pit of nude mice. Tumor volume was monitored every seven days, and the formula was as follows: *V* = (Length × Width^2^) × 0.5, nude mice were sacrificed after four weeks, and tumor size was recorded after anesthesia with isoflurane, nude mice suffered from cervical dislocation to death. Animal experiments were performed by animal care guidelines and approved by the Ethics Committee.

## Results

### Identification of tryptophan-related lncRNAs in LUAD

**Fig. 1:**
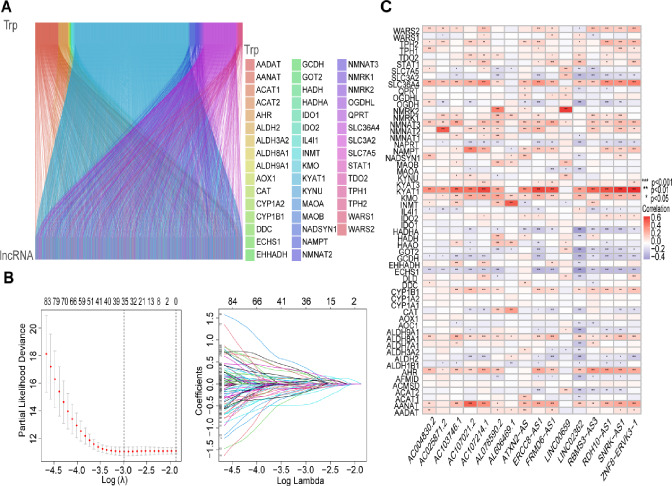
Identification of tryptophan metabolism gene-related lncRNAs and construction of prognostic models. **A** Sankey diagram showing co-expression of tryptophan metabolism-related genes and tryptophan metabolism-related lncRNAs. **B** The selection process of the optimal cross-validation parameter λ in the LASSO model and the trajectory plot of each variable. **C** Correlation heatmap showing the relationship between tryptophan metabolism-related lncRNAs and tryptophan metabolism-related genes. Red color indicates positive correlation and blue color indicates negative correlation

A total of 16,876 lncRNAs were identified in the TCGA-LUAD database. 59 tryptophan metabolism-related genes were obtained from the GSEA database (Table S1), and a network of co-expression of tryptophan metabolism-related lncRNAs was constructed to identify the lncRNAs related to tryptophan metabolism.2578 final screened tryptophan metabolism-related lncRNAs (|cor|> 0.4, *P*-value < 0.001) (Fig. 1A). Cox regression analysis was used to initially screen 137 target lncRNAs associated with the prognosis of LUAD patients (Table S2), and a forest plot was drawn (Fig.S1).

### Prognosis-based risk modeling

Further screening and analyses were then performed by LASSO regression to reduce the overfitting of the data. Thirty-five tryptophan metabolism-associated lncRNAs with high prognostic values were identified, and lasso regression coefficient profiles were plotted (Fig. 1B). Finally, multivariate Cox regression analysis was used to identify 16 tryptophan metabolism-related lncRNAs with prognostic value in LUAD patients, and the LUAD prognostic model was constructed Riskscore = (– 0.786293899438286*RBMS3-AS3) + (0.729525271199102*AC107214.1) + (– 0.594981659042687*AL078590.2) + (– 0.488362224373613*ATXN2-AS) + (– 1.26851214948258*ERCC8-AS1) + (0.691321177315073*AC004830.2) + (0.396799898336932*AC107021.2) + (– 0.366051764081276*RDH10-AS1) + (– 0.869907297975532*SNRK-AS1) + (0.448278641184027*LINC00659) + (1.04921620930125*ZNF8-ERVK3-1) + (0.743359529448505*AL606469.1) + (0.550402175045129*FRMD6-AS1) + (– 0.839764551688578*AL606469.1) + (– 0.352579344066772*LINC02362) + (– 0.61899293624872*AC025871.2). The associated heatmap also shows the relationship between tryptophan metabolism -related genes and lncRNAs (Fig. 1C).

### Evaluation and validation of LUAD lncRNA signature associated with tryptophan metabolism

**Fig. 2:**
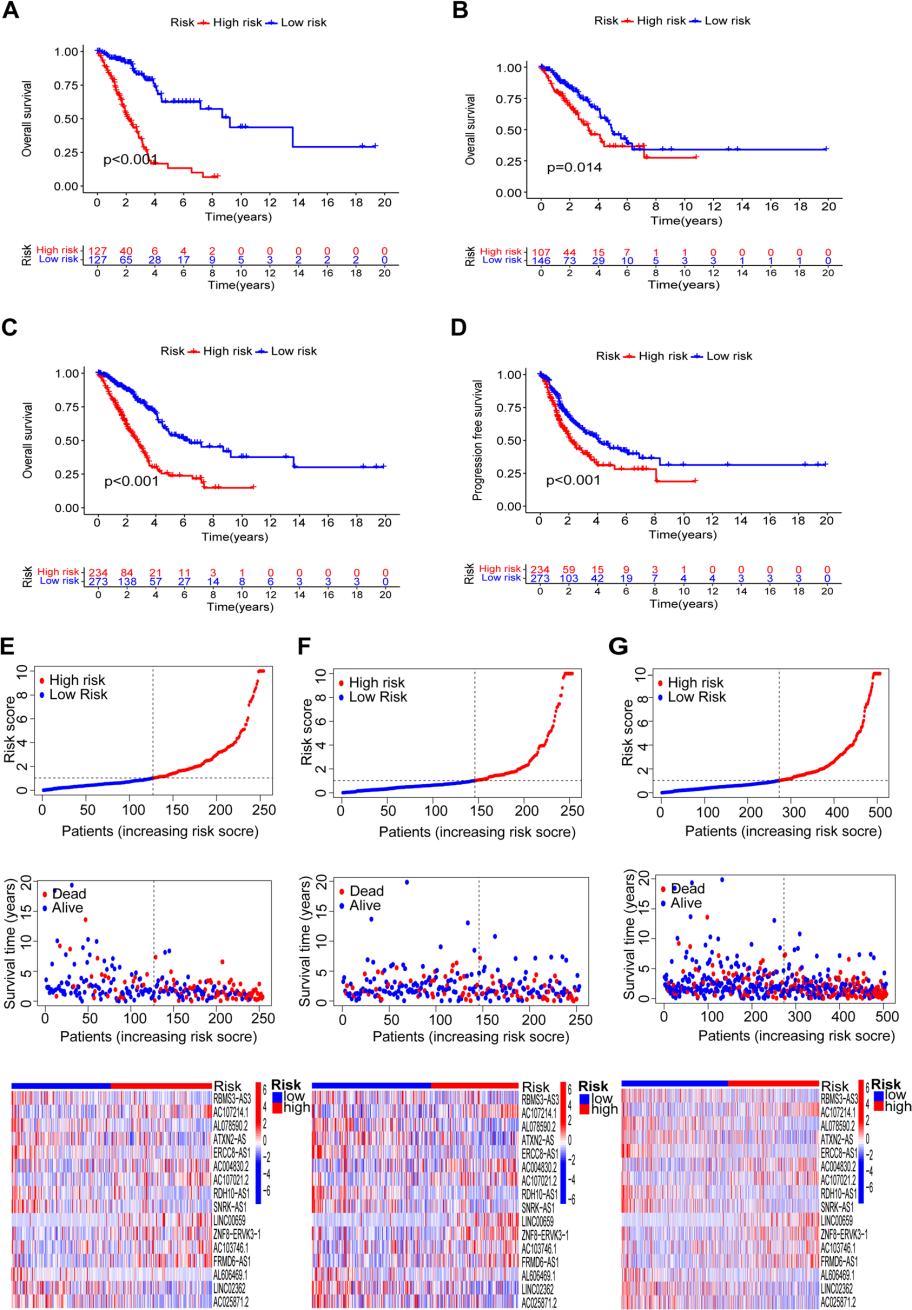
Kaplan–Meier survival analysis of patients in the high- and low-risk groups and 16 lncRNA risk score maps, survival status maps, and heat maps. **A** Training set OS. **B **Test set OS. **C** Overall set OS. **D** Overall set PFS. **E** Training set. **F** Test set. **G** Overall set

Based on the median risk score, lung adenocarcinoma patients were categorized into high-risk and low-risk groups, and OS and PFS were compared. OS and PFS were lower in the high-risk group than in the low-risk group in the training set, validation set, and overall set (Fig. 2A–D). According to the risk score and survival status display, we found that mortality increased with a higher score (Fig. 2E–G). Heatmap showed the expression of 16 tryptophan metabolism lncRNAs in high and low-risk groups.LINC00659, ZNF8-ERVK3-1, and FRMD6-AS1 were high-risk lncRNAs, while ERCC8-AS1,ATXN2-AS,RDH10-AS1,and LINC02362 were low-risk lncRNAs (Fig. 2E–G).

### Independent analysis of prognostic factors

**Fig. 3:**
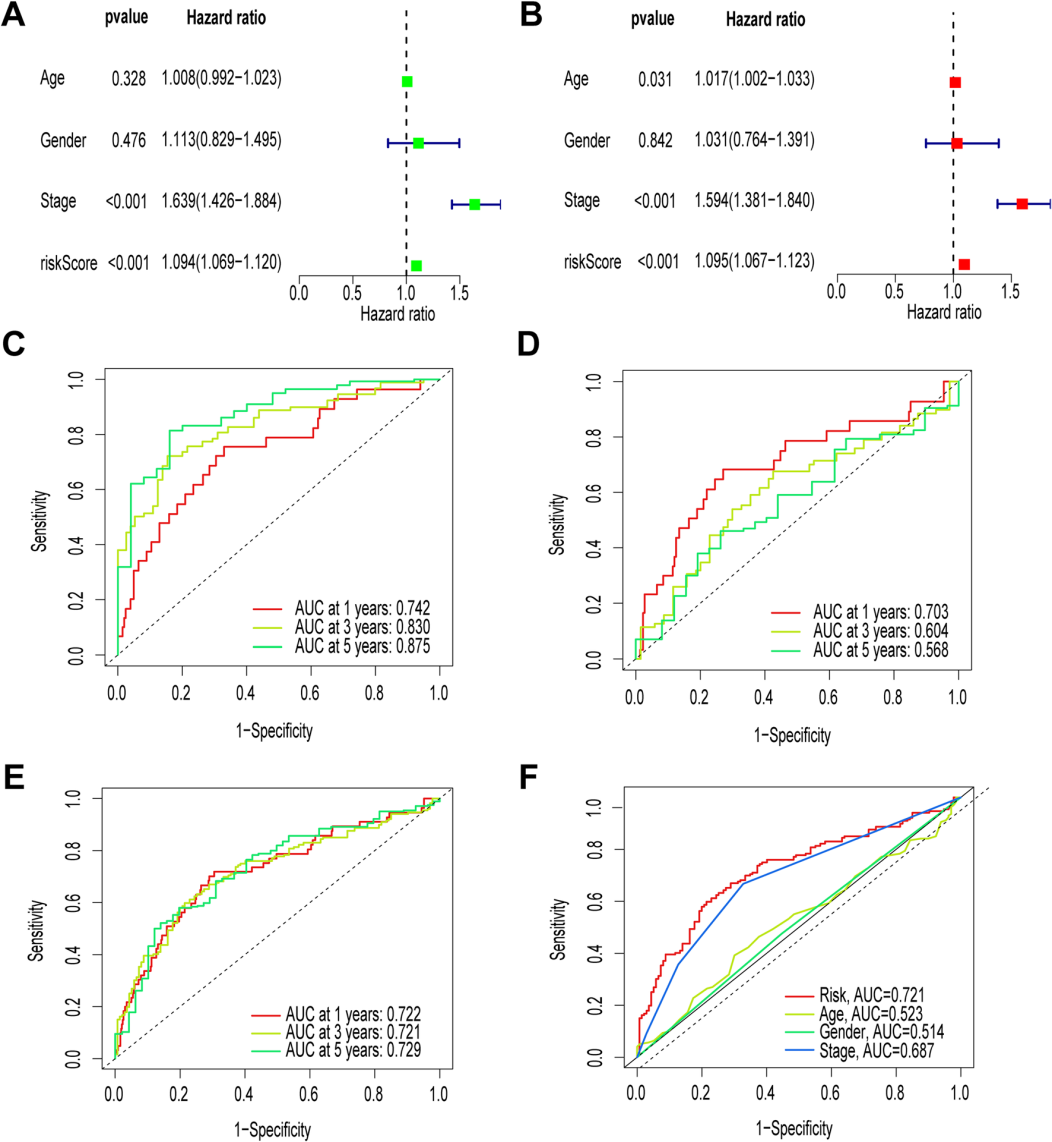
Independent predictive value of risk models. **A**, **B** Univariate Cox and multivariate Cox analyses to assess model independence from other clinical parameters. **C**–**E** ROC curves for risk scores at 1, 3, and 5 years for the training set and test set, and overall set. **F** Showing that risk scores accurately predict survival better than common clinical parameters

The results of univariate and multivariate COX regression analyses showed that the risk score could be used as an independent prognostic indicator for the prognosis of patients with LUAD (all *P* < 0.001) (Fig. 3A, B). In addition, we used ROC curves to assess the predictive accuracy of risk scores. The AUC values for the 1-, 3-, and 5-year risk scores for the training set were 0.742, 0.830, and 0.875, respectively, and the AUC values for the 1-, 3-, and 5-year values for the test set were 0.703, 0.604, and 0.568, respectively (Fig. 3C–E). Next, we performed a series of analyses based on clinical characteristics, and ROC analyses showed that risk scores had more substantial prognostic power than other clinical characteristics (Fig. 3F).

### Construction and validation of column-line diagrams

**Fig. 4:**
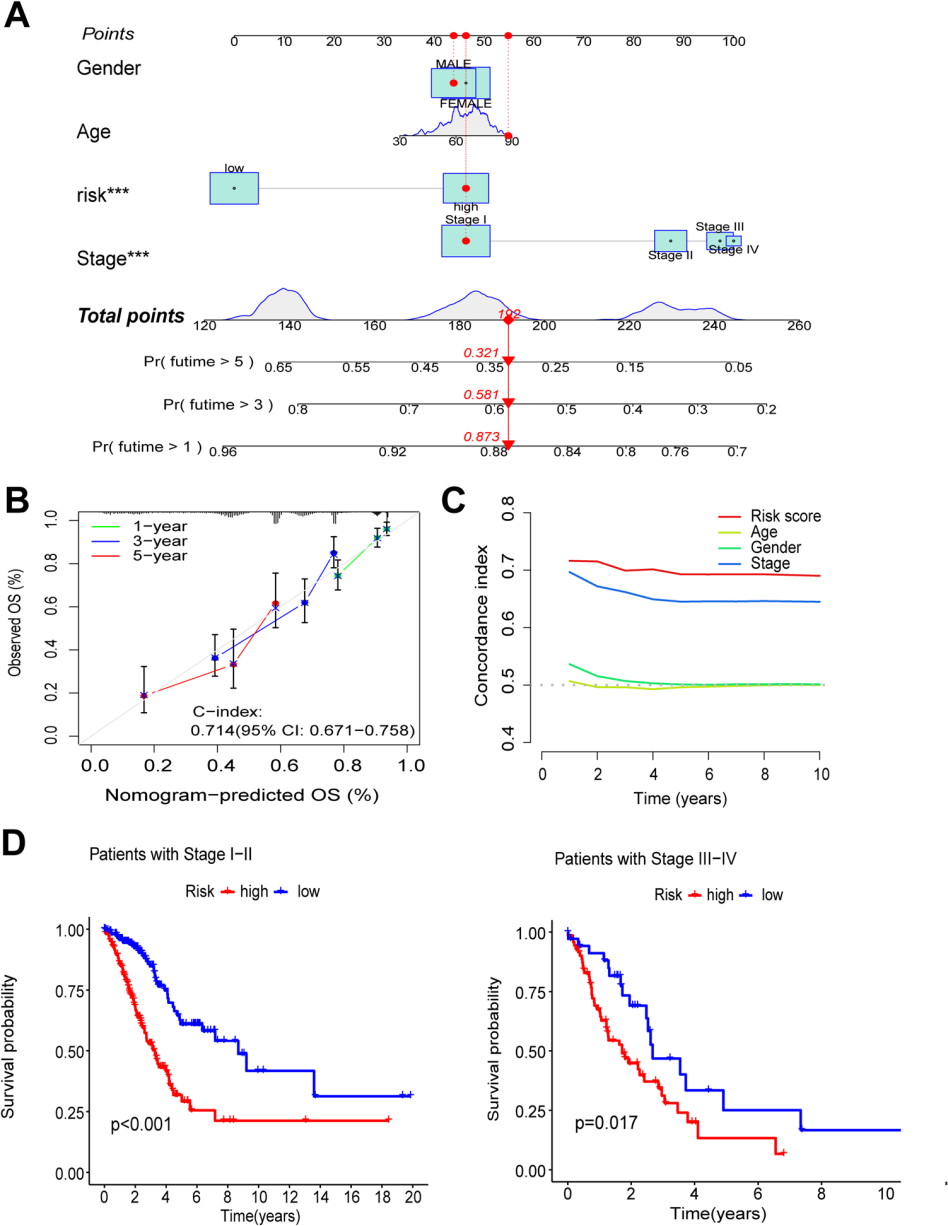
Creation of a column-line graph for predicting prognosis in patients with LUAD. **A** Nomogram survival prediction for LUAD patients with risk scores. **B** nomogram correction plot. **C** C-index curve of the risk model. **D** Risk score applied to LUAD patients with stages I–II and III–IV

Column line graphs were constructed for LUAD patients based on gender, age, risk score, and clinical stage (Fig. 4A). The calibration curves showed that the predicted OS of the 1-, 3-, and 5-year column-line diagrams were generally consistent with the corresponding observed OS of LUAD patients (Fig. 4B). We also found that the C-index value of the risk score was higher than other clinical characteristics (Fig. 4C). We further analyzed the significant difference (*P* < 0.05) in OS between patients in the high-risk and low-risk groups at different stages (Stages I–II and III–IV) (Fig. 4D, E), which suggests that the model has high predictive accuracy and can be used to compare the survival of patients at different stages. Finally, we performed PCA to observe the distribution of all genes, tryptophan metabolism-related genes, tryptophan metabolism-related lncRNAs, and risk lncRNAs in LUAD patients, and the results showed a clear distribution, suggesting that these lncRNAs can be reliably used to construct the model (Fig.S2).

### Validation of risk profiles in GEO cohorts

**Fig. 5:**
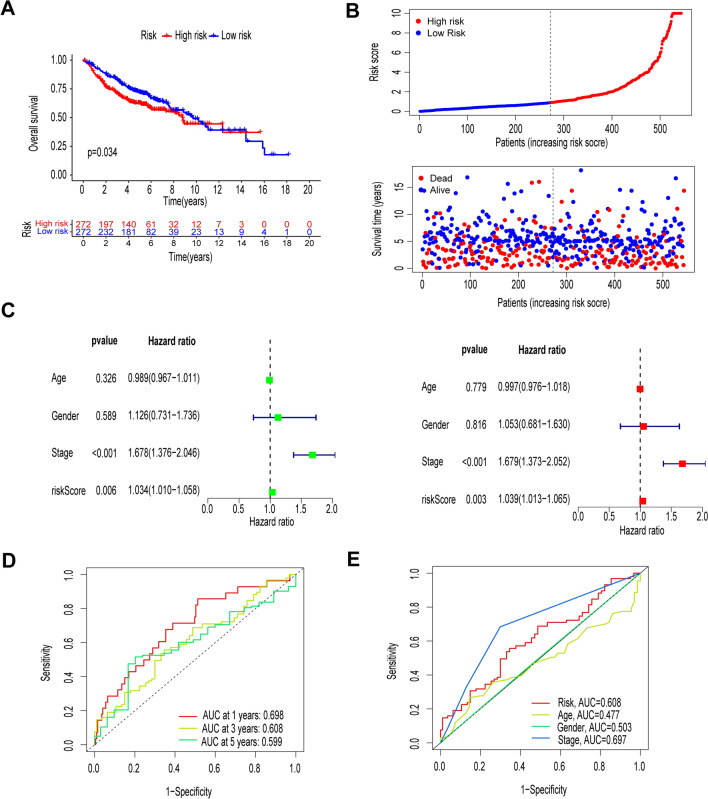
Validation of risk profiles in GEO cohorts. **A** Kaplan–Meier survival analysis of overall survival (OS) in the GEO cohorts. **B** Risk score maps, survival status maps. **C** Univariate and multivariate Cox analyses assessed the independence of the model from other clinical parameters. **D** ROC curves for risk scores at 1, 3, and 5 years. **E** ROC curves for clinical characteristics and risk scores. CI, confidence interval; HR: risk ratio; OS, overall survival; ROC, subject operating characteristic curve

Kaplan–Meier survival analysis showed that patients in the high-risk group had poorer OS than those in the low-risk group (Fig. 5A). Risk scores and survival outcomes are shown in Fig. 5B. We also included risk models and clinical characteristics in univariate and multivariate Cox regression analyses (Fig. 5C). Univariate Cox regression analysis showed that risk score was an independent prognostic factor (HR: 1.034, 95% CI 1.010–1.058, *P* < 0.05). After adjusting for potential confounders in the multivariate Cox regression analysis, the correlation between risk score and OS remained significant (HR : 1.039; 95% CI 1.013–1.065, *P* < 0.05). ROC curves revealed reliable predictive efficacy of our model in the GEO cohort (Fig. 5D). The AUC for 1 year, 3 years, and 5 years were 0.698, 0.608, and 0.599, respectively. Next, our ROC analysis based on clinical characteristics showed that the risk score also had good prognostic power (Fig. 5E).

### Gene set enrichment analysis in LUAD patients

**Fig. 6:**
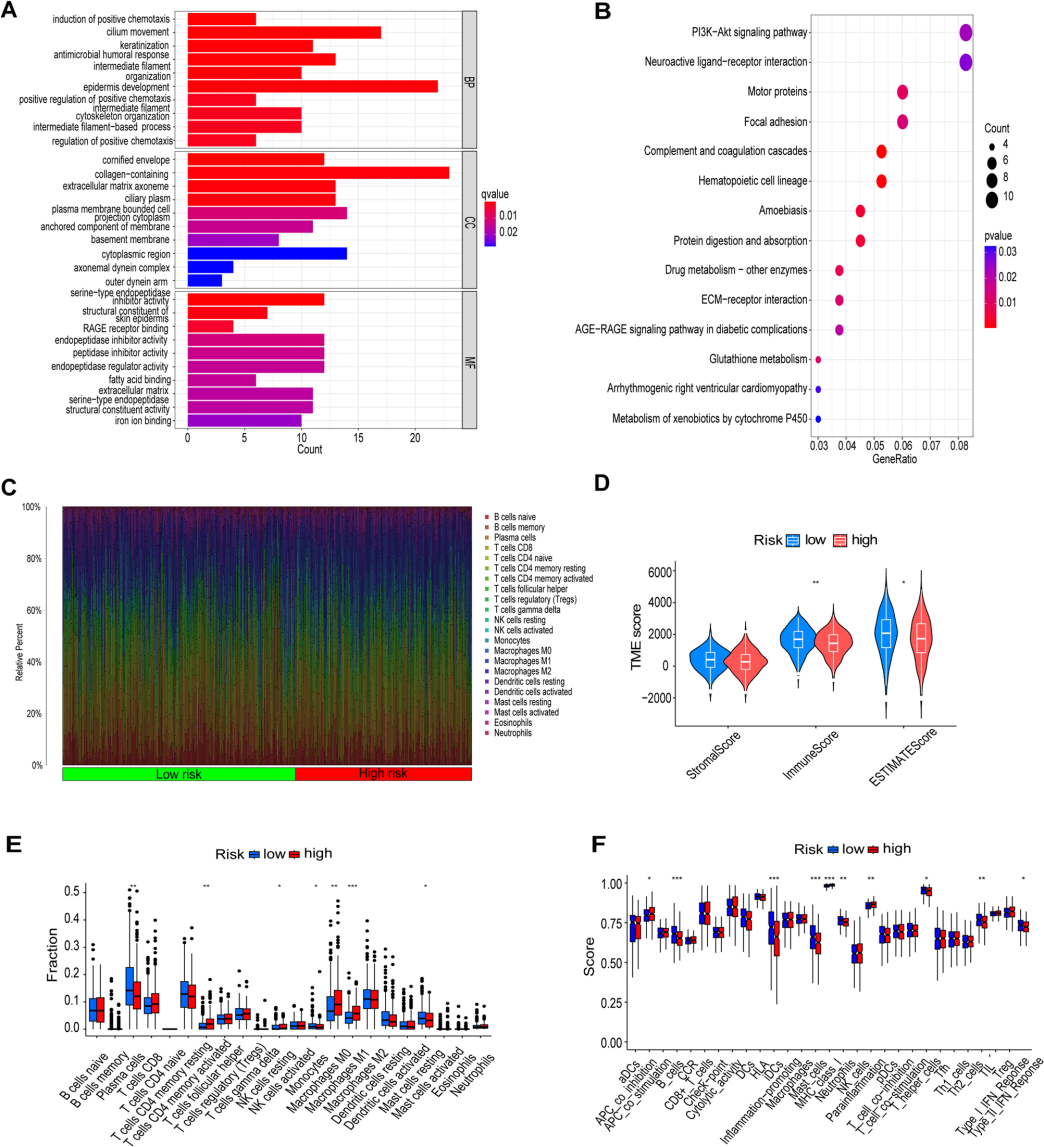
GO and KEGG analyses and TME characteristics of high and low-risk groups. **A** Barplot of the first 10 GO-enriched conditions. **B** Bubble plot of the first 30 KEGG-enriched terms. **C**, **E** Proportion of immune cells between the two groups. **D** Stromal scores, immune scores, and estimated scores between the two groups. **F** Differences in immune-related functions between the two groups. Asterisks indicate statistical significance, *, *P* < 0.05; **, *P* < 0.01; ***, *P* < 0.001

551 DEGs were identified between the high- and low-risk groups (Table S3). To reveal the biological pathways of lncRNAs associated with tryptophan metabolism, GO functional enrichment and KEGG pathway enrichment were analyzed based on the DEGs between the high- and low-risk groups. The GO analysis showed that tryptophan metabolism-associated lncRNAs in biological processes (BP) were mainly concentrated in epidermis development, and cellular components (CC) were mainly concentrated in collagen-containing, extracellular matrix, and cytoplasmic region categories. Categories, etc. Molecular functions (MF) mainly focused on endopeptidase inhibitor activity and peptidase inhibitor activity categories (Fig. 6A). The KEGG pathway indicated that tryptophan metabolism-related lncRNAs were involved in tryptophan metabolism. The KEGG pathway indicated that lncRNAs related to tryptophan metabolism were involved in Complement and coagulation cascades, Hematopoietic cell lineage, and Amoebiasis (Fig. 6B).

### Tumor immunoscape based on risk modeling

To further explore the relationship between tryptophan metabolism-associated lncRNAs and the tumor microenvironment in LUAD patients, we determined the landscape of immune cell infiltration in all LUAD patients from the TCGA database using the CIBERSORT algorithm. The proportions of each type of immune cell (Fig. 6C) are shown. To determine the difference in infiltrating immune cells between the high- and low-risk groups, we assessed this by the immunity score (immune cell infiltration in tumor tissue) and the estimated score (sum of stromal and immunity scores for individual cases), which were both significantly higher (*P* < 0.01) in the low-risk group (Fig. 6D). In addition, we compared the proportion of each immune cell between the high-risk and low-risk groups and found significant differences in Plasma cells, T cells CD4 memory activated, M1 and M0 macrophages, Monocytes, Mast cells resting, and NK cells resting between the two groups (Fig. 6E). We further explored the relationship between risk scores and immune-related features, and ssGSEA analysis showed that higher risk scores were significantly associated with reduced levels of most immune-related features (Fig. 6F), including immune cell infiltration (e.g., B cells, iDCs, Mast_cells, Neutrophils, T_helper_cells, and TIL).

### Tumor mutation load analysis and prediction of response to drug therapy

**Fig. 7:**
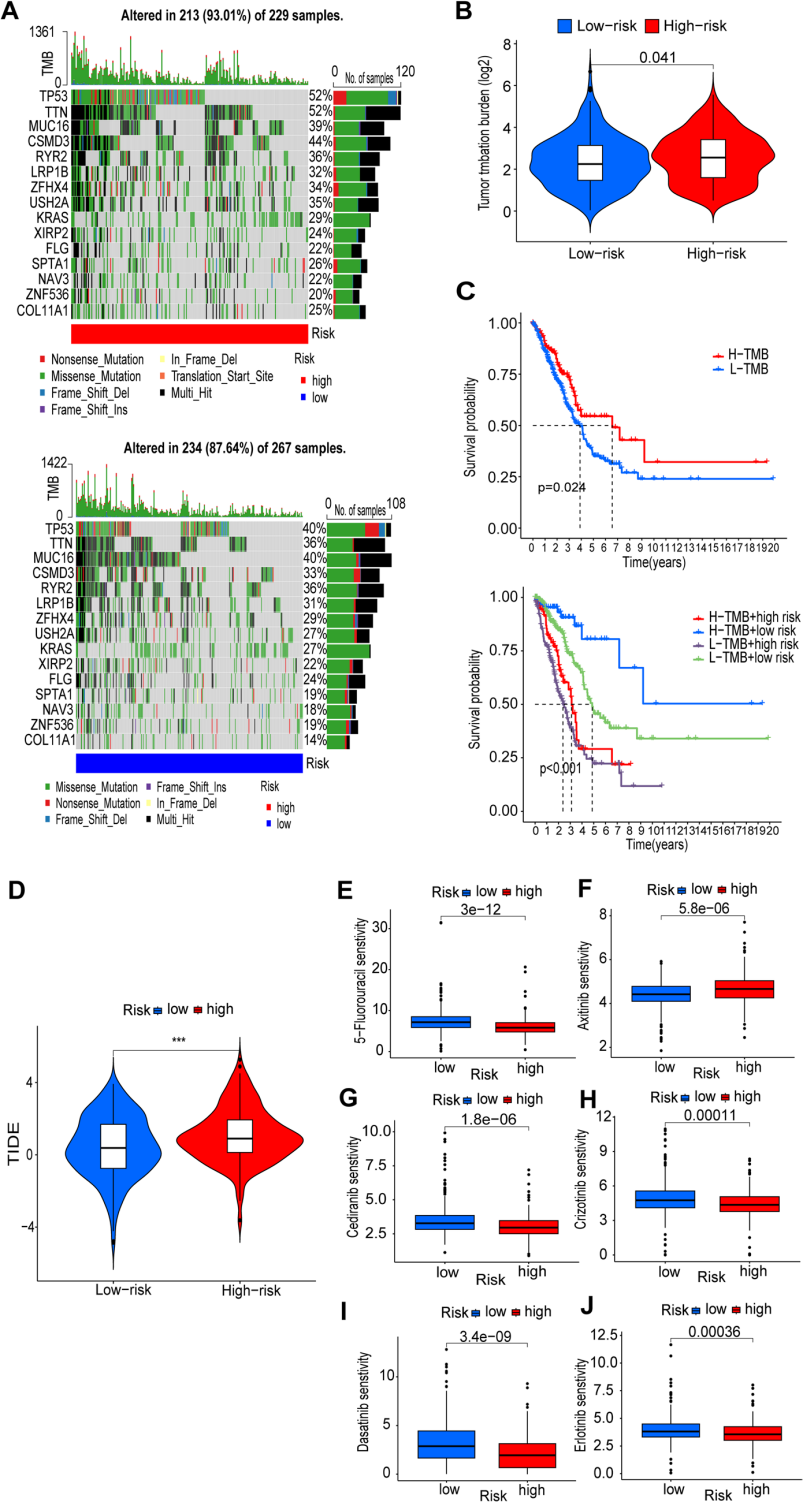
Mutation analysis of tumor somatic cells and prediction of response to drug therapy. **A** Waterfall plots showing the top 15 mutated genes in LUAD in the high-risk group (213 samples) and low-risk group (234 samples). **B** Differences in TMB between the two groups. **C** KM curves of OS in the high and low TMB groups. KM curves of OS in patients stratified by risk score and TMB subgroups. *TMB* tumor mutational load, *H* high, *L* low, *LUAD* lung adenocarcinoma, *OS* overall survival. **D** Correlation between risk scores and response to immunotherapy. **E**–**J** Drug sensitivity of 5-fluorouracil, axitinib, cediranib, crizotinib, dasatinib, and erlotinib was observed. * indicates statistical significance, *, *P* < 0.05; **, *P* < 0.01; ***, *P* < 0.001

We used the maftools algorithm to observe mutations in the high-risk and low-risk groups, and the high-risk group showed a wider range of somatic mutations than the low-risk group (TP53: low risk, 40%; high risk 52%, TTN: low risk, 36%; high risk, 52%, and CSMD3: low risk, 33%; high risk, 44%) (Fig. 7A). There was a difference in TMB difference between high- and low-risk groups (*P* < 0.05) (Fig. 7B). Kaplan–Meier survival curves showed that patients with higher TMB had better OS than those with lower TMB. The combination of risk scores and TMB showed greater prognostic value for patients with LUAD (Fig. 7C).

The risk of tumor immune escape was calculated using the TIDE algorithm. The results showed that the TIDE score was higher in the high-risk group than in the low-risk group, indicating a higher probability of immune escape (*P* < 0.001) (Fig. 7D). We further explored the potential effective therapeutic agents for LUAD patients using the "oncoPredict" R package. Among the drugs commonly used in the treatment of LUAD, 5-fluorouracil, cediranib, crizotinib, dasatinib, and erlotinib were found to have lower IC50 values (50% inhibition of cell growth) in the high-risk group of patients, suggesting that these drugs may be more effective in high-risk patients. However, low-risk patients may benefit more from axitinib (Fig. 7E–J).

### ZNF8-ERVK3-1 expression was significantly elevated in LUAD tissues and cells

**Fig. 8:**
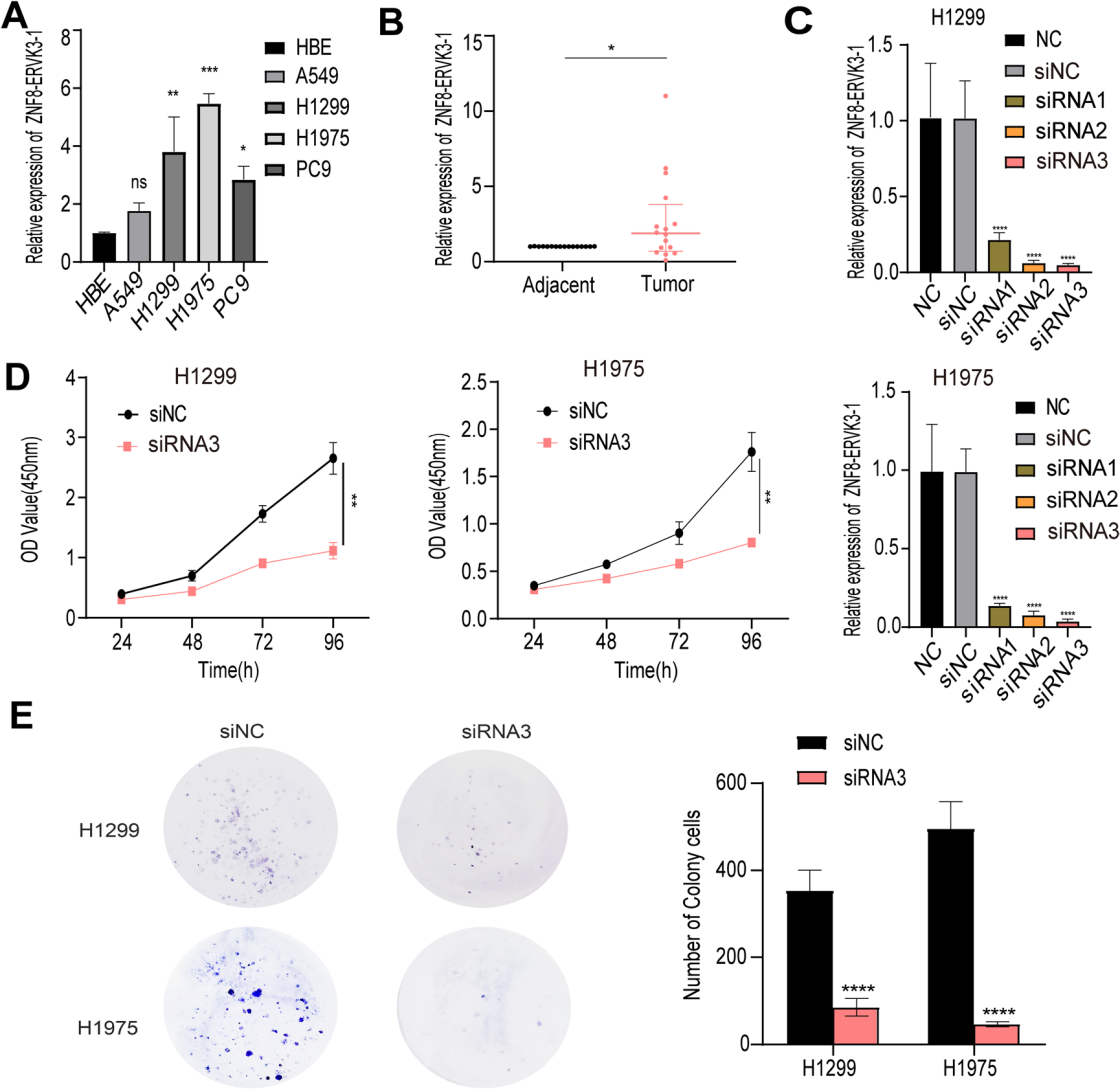
ZNF8-ERVK3-1 expression was upregulated in LUAD tissues and cells. ZNF8-ERVK3-1 knockdown inhibited tumor cell proliferation, migration, and invasion. **A** The expression of ZNF8-ERVK3-1 in LUAD cells (A549, H1299, H1975, PC9) and HBE was determined by qRT-PCR. *, *P* < 0.05; **, *P* < 0.01; ***, *P* < 0.001. **B** ZNF8-ERVK3-1 expression was detected in 16 pairs of LUAD tissues and adjacent non-tumor tissues by qRT-PCR. **C** The efficiency of ZNF8-ERVK3-1 knockdown in H1299 and H1975 cells transfected with siRNA1 / 2 / 3 was determined by both qRT-PCR. **D**, **E** The viability and proliferation ability of H1299 and H1975 cells transfected with siRNA3 were determined by CCK-8 and clone formation assay

Differential expression of ZNF8-ERVK3-1 in LUAD cell line and normal human bronchial epithelioid cell line (HBE) was verified by qRT-PCR assay, and it was found that the expression of LUAD cell line was higher than that of HBE (Fig. 8A). Further analysis of the RNA levels of ZNF8-ERVK3-1 in lung adenocarcinoma tissues and paired normal tissues showed that the expression of ZNF8-ERVK3-1 was significantly higher in LUAD tissues (Fig. 8B).

### ZNF8-ERVK3-1 knockdown inhibits tumor cell proliferation, migration, invasion, and G1 phase inhibits and promotes apoptosis

H1299 and H1975 cells had high expression of ZNF8-ERVK3-1, so H1299 and H1975 cells were transfected using si-ZNF8-ERVK3-1, and the knockdown efficiency was verified by qPCR (Fig. 8C). siRNA3 was selected to perform a series of functional experiments. CCK-8, clone formation experiments showed that knockdown of ZNF8-ERVK3-1 gene resulted in growth retardation of H1299 and H1975 cells (Fig. 8D, E). The complete original image of clone formation experiments is supplemented in Fig.S3.

**Fig. 9:**
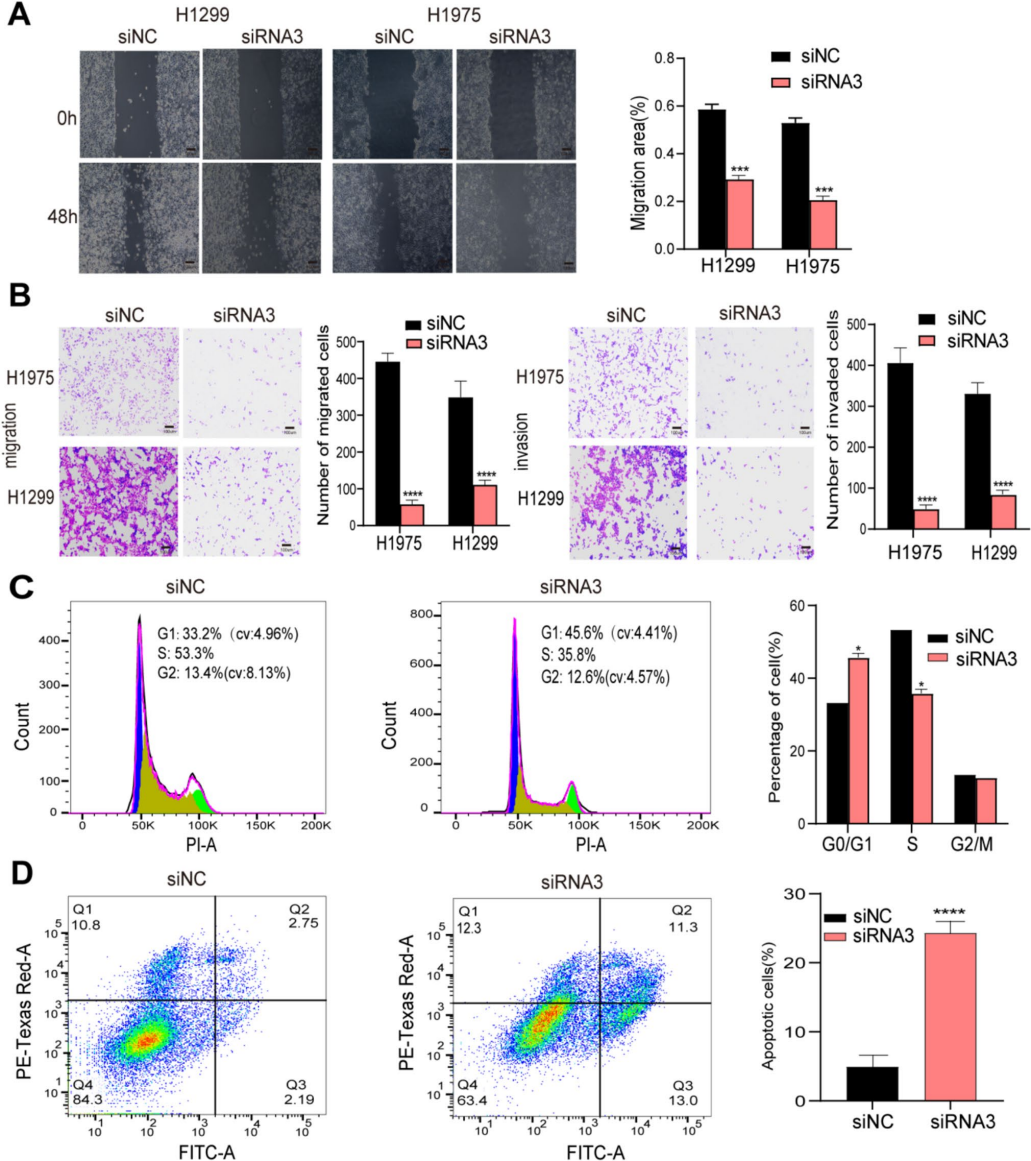
ZNF8-ERVK3-1 knockdown inhibited tumor cell migration, invasion and G0/G1 cell cycle arrest and apoptosis were increased. **A** Wound healing assay examining the mobility of H1299 and H1975 cells transfected with siNC and siRNA3. **B** The number of H1299 and H1975 cells transfected with siNC and siRNA3 migrated and invaded were assessed by transwell assay. **C** The effect of ZNF8-ERVK3-1 knockdown on H1975 cells cycle was detected using Cell Cycle and Apoptosis Analysis Kit. **D** The effect of ZNF8-ERVK3-1 knockdown on apoptosis of H1975 cells was detected using Annexin V Apoptosis Detection Kit. *P < 0.05, **P < 0.01, ****P < 0.0001

Wound healing and transwell assays showed that ZNF8-ERVK3-1 knockdown resulted in a significant decrease in cell migration (Fig. 9A) and a dramatic decrease in their invasive ability (Fig. 9B). In addition, we applied flow cytometry to explore whether ZNF8-ERVK3-1 knockdown leads to LUAD cell cycle arrest and increased apoptosis. In H1975 cells, the proportion of cells in the G0/G1 phase was significantly increased in both the si-ZNF8-ERVK3-1 group compared with the si-NC group, while the proportion of cells in S and G2/M phases was decreased (Fig. 9C). We further found that the apoptosis rate was significantly increased after ZNF8-ERVK3-1 knockdown (Fig. 9D).

### ZNF8-ERVK3-1 promotes tumorigenesis in vivo

**Fig. 10:**
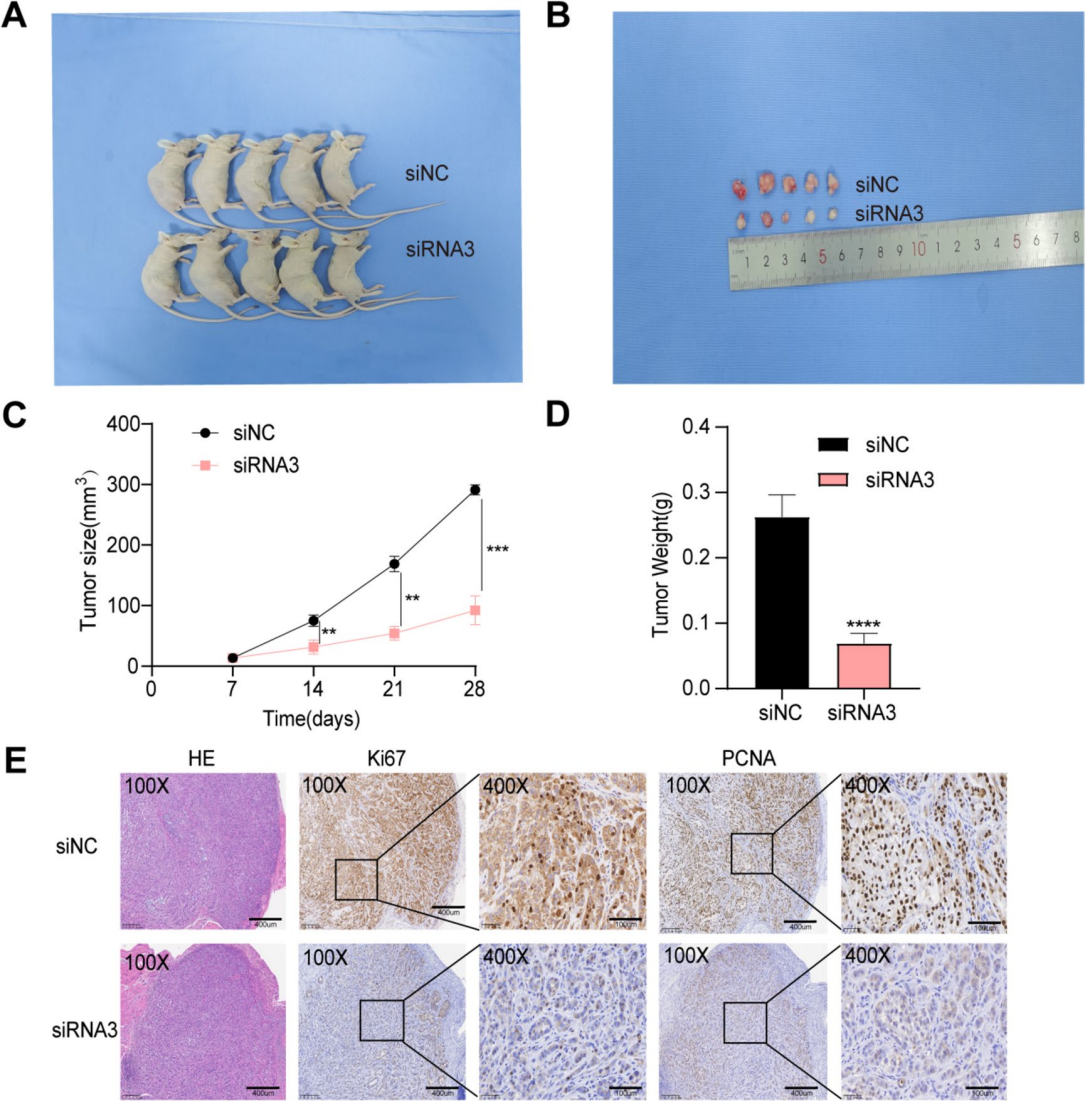
ZNF8-ERVK3-1 promotes in vivo tumorigenesis. **A**, **B** Xenograft tumors in nude mouse models. **C** Tumor size. **D** Tumor weight.** E** The expression of ki67 and PCNA in siNC and siRNA3 subcutaneous xenografts was analyzed by immunohistochemistry. *P < 0.05, **P < 0.01

A xenograft nude mouse model was established to elucidate the role of ZNF8-ERVK3-1 in LUAD in vivo. Tumor changes were closely monitored after injection of H1975 cells from transfected siNC and siRNA3. The results showed that ZNF8-ERVK3-1 knockdown inhibited tumor growth, i.e., a significant reduction in tumor size and weight (Fig. 10A–D). Immunohistochemical analysis showed that the expression level of ki67 and PCNA, which are closely related to tumor proliferation, were significantly lower in the siRNA3 group than in siNC (Fig. 10E).

## Discussion

Lung adenocarcinoma is one of the most common pathological types of lung cancer. Despite advances in the fields of surgery, radiotherapy, chemotherapy, and immunotherapy, the overall survival of LUAD remains poor (Allemani et al. 2015). Therefore, we want to continue to explore novel molecular biomarkers for individualized prediction of LUAD prognosis and provide new targets for future LUAD treatment. An increasing number of lncRNAs have been shown to play critical roles in the onset and progression of LUAD (Qu et al. 2021; Loewen et al. 2014). For example, lncRNA UPLA1 can be a prognostic marker to promote lung adenocarcinoma progression through Wnt/β-linker protein signaling (Han et al. 2020). Jiang et al. found that lncRNA HCP5 acts as a novel regulator in the TGFβ/SMAD signaling pathway to promote LUAD tumor growth and metastasis (Jiang et al. 2019). Recently, tryptophan metabolism was found to be closely related to regulating immunity and tumorigenesis (Kwiatkowska et al. 2021). Meanwhile, the tryptophan metabolic pathway and its metabolites have multiple functions in lung cancer pathogenesis, including regulating the tumor microenvironment and promoting immunosuppression and drug resistance (Li and Zhao 2021). In this study, we successfully established a prognostic risk profile based on tryptophan metabolism-associated lncRNAs for predicting overall survival in LUAD patients. In addition, we preliminarily validated the oncogenic role of ZNF8-ERVK3-1 in LUAD. We found that inhibition of ZNF8-ERVK3-1 may inhibit the proliferation, migration, and invasion of LUAD cells and can also lead to cellular G0/G1 phase cycle blockage and increased apoptosis. In vivo experiments showed that ZNF8-ERVK3-1 promoted LUAD tumorigenesis.

Pearson correlation analysis showed that 2578 lncRNAs were significantly associated with 60 tryptophan metabolism-related genes (*P* < 0.001). By univariate Cox regression analysis, 137 lncRNAs were identified as independent prognostic factors for LUAD (*P* < 0.05). Finally, 16 lncRNAs were identified by multivariate Cox regression analysis to establish prognostic models, which were accurate for predicting OS and PFS in LUAD. Overexpression of RBMS3-AS3 inhibited cell proliferation, migration, invasion, and angiogenesis as well as tumorigenicity of prostate cancer, and RBMS3-AS3 acted as a miR-4534 sponge to inhibit cell proliferation, migration, invasion, and angiogenesis. Inhibits prostate cancer development by up-regulating VASH1 (Jiang et al. 2020). Li et al. found that ATXN2-AS may be associated with spinal cerebellar ataxia type 2 (SCA2) and amyotrophic lateral sclerosis (ALS) (Li et al. 2016). ERCC8-AS1 and RDH10-AS1 were markedly upregulated in osteosarcoma tissues, which may serve as biomarkers for osteosarcoma and potential therapeutic targets (Rothzerg et al. 2021). LINC00659 as an oncogene, Sheng et al. found that LINC00659 could promote gastric carcinogenesis by promoting SUZ12 expression (Sheng et al. 2020). Another group found that cancer-associated fibroblast (CAF)-derived exosome LINC00659 promotes colorectal cancer cell proliferation, invasion, and migration through the miR-342-3p / ANXA2 axis (Zhou et al. 2021). Wu et al. found that FRMD6-AS1 as necrotic apoptosis-associated lncRNA was significantly elevated in lung adenocarcinoma cells and tissues (Wu et al. 2022). We then used ROC analysis to assess the predictive performance of the risk model we constructed. Our model's AUC of the ROC curves for 1-, 3- and 5-year OS were 0.742, 0.83, and 0.87, respectively. In addition, the AUC of the ROC curves of our model were more significant than the conventional clinical characteristics of the patients. All LUAD samples were randomly divided into a training set (50%) and a test set (50%) to validate the confidence of our risk model. The training and test sets were divided into high-risk and low-risk groups based on the median risk score. For the training set, the overall survival of LUAD patients in the high-risk group was significantly shorter than that in the low-risk group (*P* < 0.001). Moreover, the results of the training set were similar to those of the test set (*P* = 0.014). Univariate and multivariate COX regression analyses showed that risk score and clinical stage were independent indicators affecting the prognosis of LUAD patients (*P* < 0.05). We constructed a column-line graph combining risk scores and clinical characteristics to reliably predict the prognosis of patients with LUAD. The predictive model showed the same predictive ability in the GEO-LUAD validation cohort.

We performed an immune cell infiltration analysis using CIBERSORT and examined the correlation between immune cell infiltration and risk scores.M1-type macrophages have pro-inflammatory, immunogenic, and anti-tumor properties (Ginhoux and Guilliams 2016). In our study, we found that patients with higher risk scores had higher M1 macrophage infiltration scores, suggesting that tumors in high-risk patients may have higher M1 macrophage infiltration.

TMB, defined as the number of somatic mutations per megabase, is often used as a predictive biomarker for immune checkpoint blockade in lung cancer (Fusco et al. 2021). We analyzed the TMB status of lung adenocarcinoma patients in the high-risk and low-risk groups. The high-risk group exhibited higher TMB than the low-risk group. some mutations were strongly associated with risk scores. For example, mutations in TP53, TTN, and CSMD3 were the top three mutations in the high-risk group.TP53 is the most commonly mutated gene in patients with NSCLC. The tumor-suppressor function of the p53 protein is reversed in TP53-mutated individuals, who exhibit pro-cancer effects and have a poorer prognosis (Bykov et al. 2018). TTN is associated with increased TMB in a variety of solid tumors and is closely related to the objective response to ICB (Jia et al. 2019). Liu et al. found that CSMD3, a common mutated gene in lung cancer, and CSMD3 deletion resulted in increased proliferation of airway epithelial cells (Liu et al. 2012). In addition, the combination of TMB and risk modeling has brought more accurate survival analysis to patients.

TIDE is a computational method used to predict ICB response (Jiang et al. 2018). According to the TIDE prediction results, patients in the high-risk group had higher TIDE values. This finding suggests high-risk patients have a higher potential for tumor immune escape. We used the "oncoPredict" R package to investigate the potential effective therapeutic agents for LUAD patients. Drug sensitivity analyses showed that patients with high-risk scores might be more sensitive to 5-fluorouracil, cediranib, crizotinib, dasatinib, and erlotinib, a tyrosine kinase inhibitor with a broad spectrum of anti-tumor activity in non-small cell lung cancer (Nikolinakos and Heymach 2008). Studies have shown that crizotinib prolongs the survival of patients with ALK mutation-positive non-small cell lung cancer (Solomon et al. 2018). Dasatinib, a multi-targeted protein tyrosine kinase inhibitor targeting the BCR-ABL and SRC family of kinases, has been successfully used in the treatment of chronic myeloid leukemia (CML), and several studies have shown that dasatinib inhibits the lung cancer cell proliferation in vitro and tumor growth in vitro (Zhang et al. 2020, 2023; Redin et al. 2021).

Finally, we analyzed the role of ZNF8-ERVK3-1, a lncRNA associated with tryptophan metabolism, in LUAD. We verified that ZNF8-ERVK3-1 expression was significantly elevated in LUAD tissues and cells. We also explored the proliferation, migration, and invasion of ZNF8-ERVK3-1 using CCK-8, clone formation, wound healing, and Transwell assays. We found that the knockdown of ZNF8-ERVK3-1 inhibited the proliferation, migration, and invasion of LUAD cells. We also found that the knockdown of ZNF8-ERVK3-1 resulted in G0/G1 phase cycle block and increased apoptosis in LUAD cells by flow cytometry analysis. In vivo experiments further confirmed that ZNF8-ERVK3-1 promoted LUAD tumorigenesis.

Although our findings have been validated in an independent cohort, there are some limitations. First, our study is a retrospective study based on the publicly available TCGA database, and prognostic models need to be validated in prospective studies for clinical use. Second, the underlying mechanisms of how these lncRNAs affect tryptophan metabolism remain unknown. Further studies are necessary to investigate the relationship between these lncRNAs and tryptophan metabolism. Finally, other cohorts have not validated the correlation between our drug sensitivity prediction and immunotherapy response.

## Conclusions

In summary, we constructed a robust prognostic model of 16 tryptophan metabolism-associated lncRNAs in lung adenocarcinoma, providing new insights for predicting the prognosis of lung adenocarcinoma patients. The prognostic risk score was strongly correlated with common clinical characteristics such as immune cell infiltration, immune-related function, TMB, and anticancer drug sensitivity, which may improve the benefit rate of patients. In conclusion, we preliminarily verified by in vitro experiments that ZNF8-ERVK3-1 promotes lung adenocarcinoma proliferation, migration, and invasion and that knockdown of ZNF8-ERVK3-1 leads to G0/G1 phase cycle blockage and increased apoptosis. In vivo experiments confirmed that ZNF8-ERVK3-1 promoted LUAD tumorigenesis. It provides a theoretical basis for individualized treatment of lung adenocarcinoma.

## Supplementary Information

Below is the link to the electronic supplementary material.

Supplementary file1 (TIF 6680 KB)

Supplementary file2 (TIF 1872 KB)

Supplementary file3 (TIF 17354 KB)

Supplementary file4 (XLSX 10 KB)

Supplementary file5 (XLSX 20 KB)

Supplementary file6 (XLSX 60 KB)

## Data Availability

Data supporting the findings of this study may be obtained from the respective authors upon reasonable request.

## Abbreviations

TCGA: The Cancer Genome Atlas
GEO: Gene Expression Omnibus
LUAD: Lung adenocarcinoma
Trp: Tryptophan
TMB: Tumor mutational load
TIDE: Tumor immune dysfunction and exclusion
IDO1: Indoleamine 2,3-dioxygenase 1
LC: Lung cancer
ICIs: Immune checkpoint inhibitors
lncRNAs: Long non-coding RNAs
MSigDB: Molecular Signatures Database
LASSO: Least absolute shrinkage and selection operator regression
OS: Overall survival
PFS: Progression-free survival
ROC: Receiver operating characteristic
AUC: Area under the curve
PCA: Principal component analysis
DEGs: Differentially expressed genes
FDR: False discovery rate
GO: Gene Ontology
KEGG: Kyoto Encyclopedia of Genes and Genomes
GDSC: Genomics of Drug Sensitivity in Cancer
qRT-PCR: Quantitative real-time polymerase chain reaction
PBS: Phosphate buffer saline
PI: Propidium iodide
