# Supplementary figures and images for: Identification and validation of tryptophan metabolism-related lncRNAs in lung adenocarcinoma prognosis and immune response
Source: J Cancer Res Clin Oncol. 2024 Apr 1;150(4):171. doi: 10.1007/s00432-024-05665-x (PMC10984901; doi:10.1007/s00432-024-05665-x)

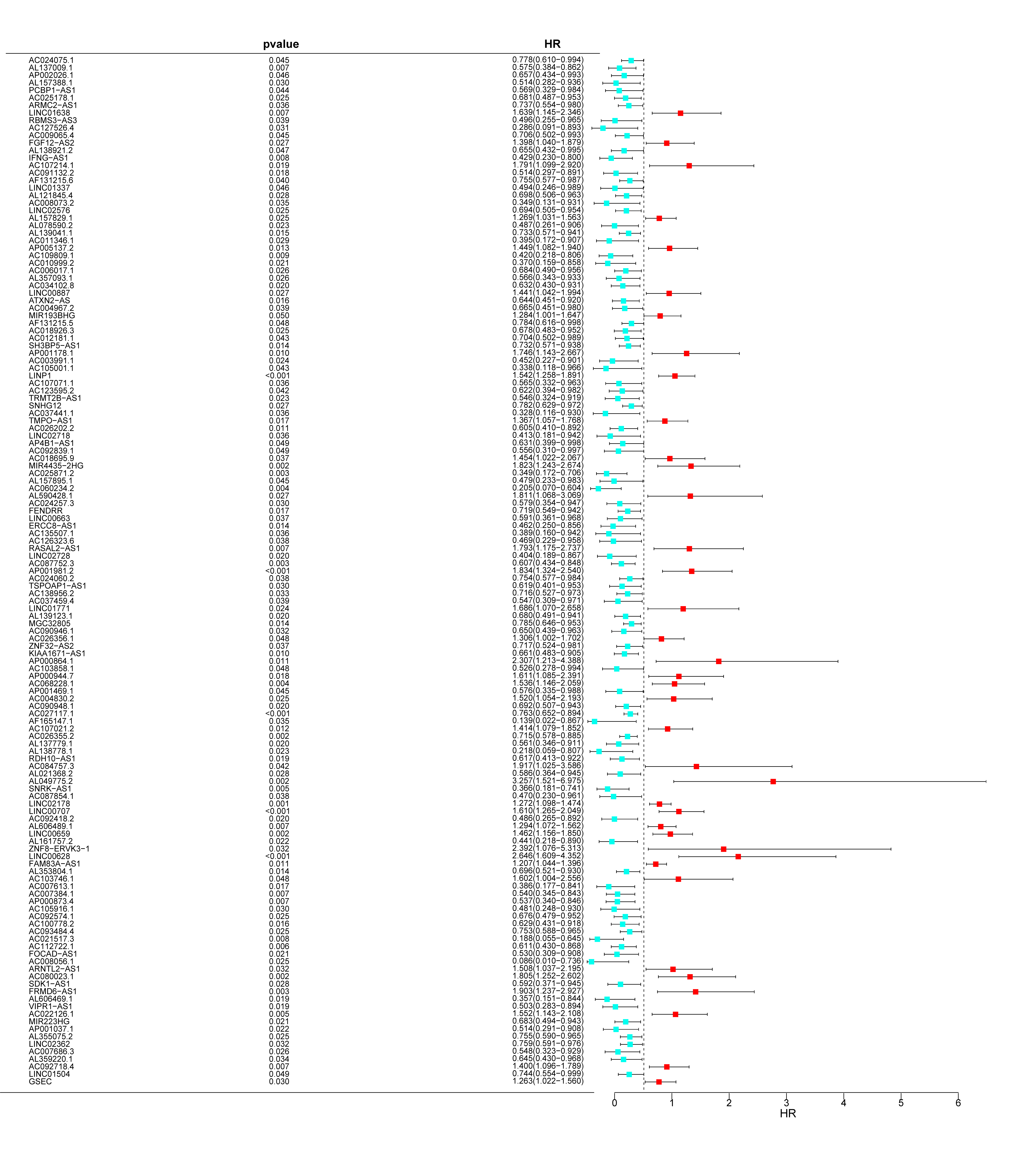

Supplement: Supplementary file 1 — Supplementary file1 (TIF 6680 KB) [file 432_2024_5665_MOESM1_ESM.tif]

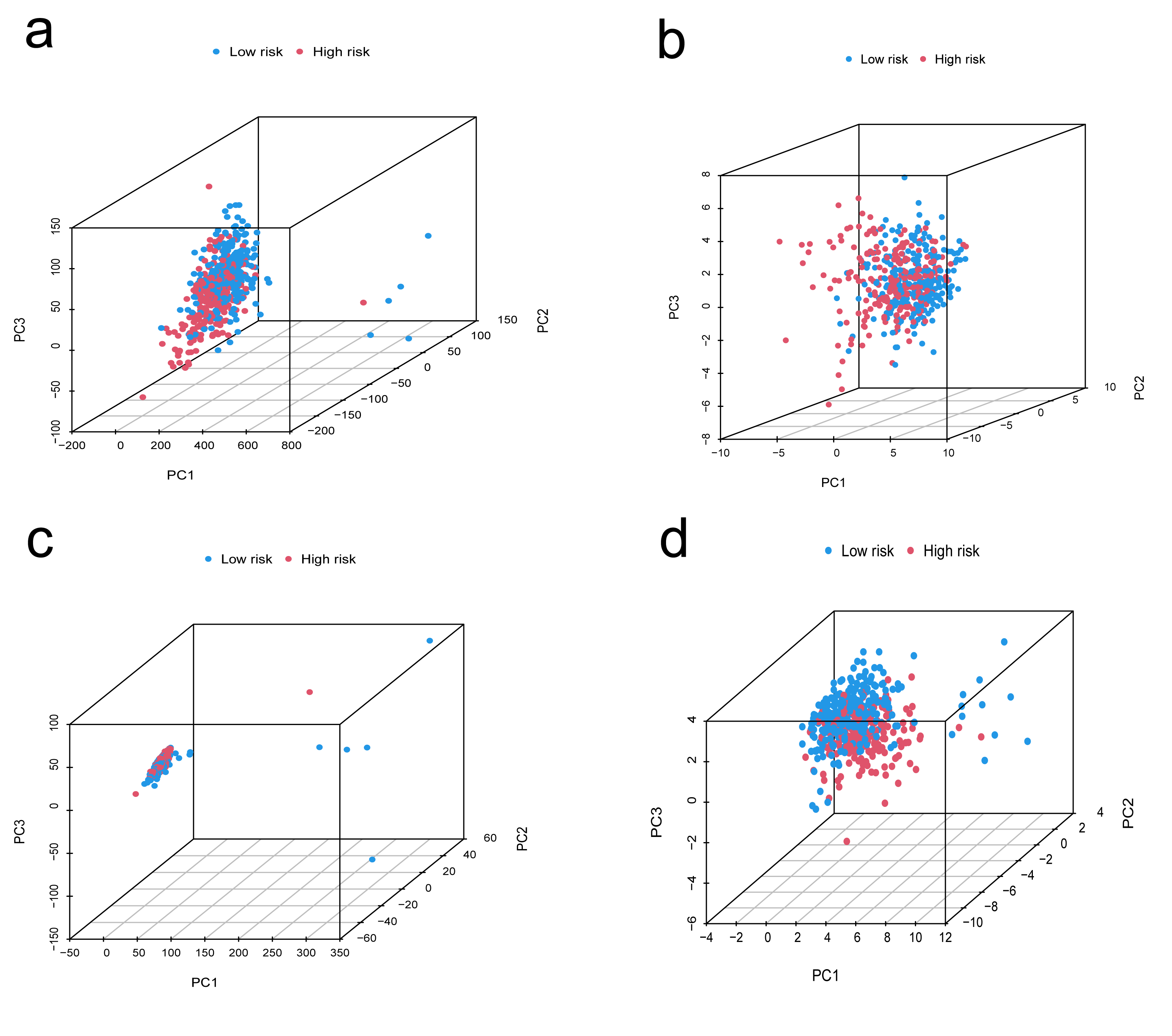

Supplement: Supplementary file 2 — Supplementary file2 (TIF 1872 KB) [file 432_2024_5665_MOESM2_ESM.tif]

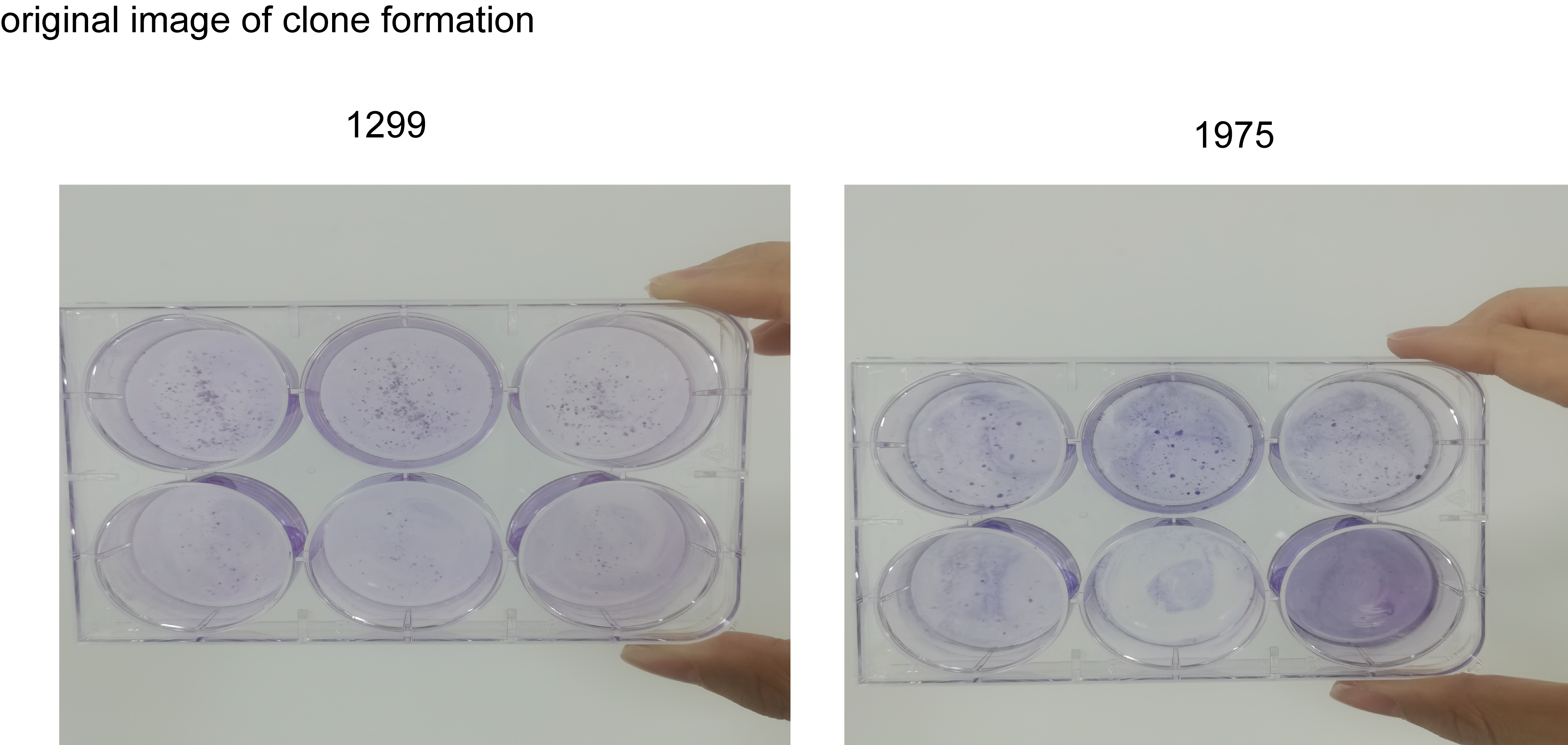

Supplement: Supplementary file 3 — Supplementary file3 (TIF 17354 KB) [file 432_2024_5665_MOESM3_ESM.tif]
